# Nausea, Vomiting, and Dyspepsia Following Solid Organ Abdominal Transplant

**DOI:** 10.7759/cureus.24274

**Published:** 2022-04-19

**Authors:** Simone A Jarrett, Kevin B Lo, Cameron Body, Joyce J Kim, Ziduo Zheng, Suprateek Kundu, Eugene Huang, Arpita Basu, Mary Flynn, Karan A Dietz-Lindo, Nikrad Shahnavaz, Jennifer Christie

**Affiliations:** 1 Internal Medicine, Einstein Medical Center Philadelphia, Philadelphia, USA; 2 Department of Gastroenterology, Wellstar Atlanta, Atlanta, USA; 3 Gastroenterology and Hepatology Division, Department of Internal Medicine, University of Michigan, Ann Arbor, USA; 4 Department of Biostatistics and Bioinformatics, Emory University Rollins School of Public Health, Atlanta, USA; 5 Department of Biostatistics, MD Anderson Cancer Center, Houston, USA; 6 Department of Biostatistics, Emory University Rollins School of Public Health, Atlanta, USA; 7 Division of Nephrology and Division of Transplant, Emory University School of Medicine, Atlanta, USA; 8 Division of Digestive Diseases, Emory University School of Medicine, Atlanta, USA

**Keywords:** dyspepsia, vomiting, nausea, solid organ transplant, gastroparesis

## Abstract

Background and objective

Multiple comorbidities may contribute to high readmission rates post-transplant procedures. In this study, we aimed to assess the rates and factors associated with hospital readmissions for dyspeptic symptoms among transplant patients.

Methods

This was a retrospective analysis of adult patients who underwent solid organ transplants at our institution. Pregnant patients or those patients with preexisting gastroparesis were excluded from the study. Readmissions associated with the International Classification of Diseases (ICD) codes for nausea/vomiting, weight loss, failure to thrive, abdominal pain, and/or bloating were included. Factors associated with 30-day and frequent readmissions (two or more) were explored.

Results

A total of 931 patients with solid organ transplants were included; 54% had undergone kidney transplants while 34% were liver transplants. Of note, 30% were readmitted within the first 30 days after discharge following transplant while 32.3% had frequent readmissions. A post-transplant upper endoscopy (EGD) was performed in 34% with food residue discovered in 19% suggesting gastroparesis. However, since only 22% of these patients had a gastric emptying study, only 6% were formally diagnosed with gastroparesis, which was independently associated with both 30-day [odds ratios (OR): 2.58, 95% confidence intervals (CI): 1.42-4.69] and frequent readmissions (OR: 6.71, 95% CI: 3.45-13.10). The presence of pre-transplant diabetes (35%) was significantly associated with a diagnosis of gastroparesis following transplant (OR: 5.17, 95% CI: 2.79-9.57). The use of belatacept (OR: 0.63, 95% CI: 0.42-0.94, p=0.023) was associated with a decrease in the odds of 30-day readmissions.

Conclusion

A significant number of patients were readmitted due to dyspeptic symptoms after solid organ transplants. Diabetes and gastroparesis were significantly associated with higher odds of readmissions while the use of belatacept appeared to be a protective factor.

## Introduction

In the decades since the first successful kidney transplant in 1954, the field of organ transplant has progressed tremendously. Solid organ transplant has been established as a life-saving treatment option for those with end-organ failures such as those in kidneys, liver, pancreas, lungs, and heart. Data from the last few years have shown that more than two million life-years were saved by solid organ transplants over a 25-year period in the United States alone and, because of this, the support for organ donation and the establishment of transplant centers has risen [1]. While this may be a life-saving treatment, these complex surgeries are typically performed on patients with multiple comorbidities, contributing to high readmission rates among post-transplant patients due to complications that are associated with transplants. Recent studies have shown that abdominal pain and gastrointestinal (GI) symptoms are the most common presentations in the emergency department in patients following a transplant procedure [2]. The incidence of such readmissions is underreported by physicians as well as in the literature, and it may adversely affect the patient quality of life and transplant outcomes.

The inpatient gastroenterology service at our quaternary referral center is frequently called on to assess post-transplant patients, particularly regarding upper GI complaints. The aim of our study was to assess the rates of hospital readmissions for dyspeptic symptoms and determine the variables associated with these common complaints in transplant patients.

## Materials and methods

Study design, participants, and data collection

This study was a single-center, retrospective analysis of all adult patients aged 18 years and above who underwent solid organ abdominal transplants at our University Hospital between June 1, 2008, and June 1, 2018. Patients with a known diagnosis of gastroparesis, those who were less than 18 years of age, and pregnant patients were excluded. Demographic and clinical factors, including age, gender, race, and medical comorbidities, were extracted from electronic medical records and documented on a standardized data collection form. Patients were included for analysis if the diagnosis for readmission was associated with the International Classification of Diseases Ninth Revision (ICD-9) or Tenth Revision (ICD-10) codes for nausea/vomiting, weight loss, failure to thrive, and/or bloating, and a detailed chart review was conducted. The frequency of readmissions for upper GI symptoms and the associated medical workup of these symptoms were assessed. This study was approved by our university's institutional review board.

Statistical analysis 

Data were summarized and presented using descriptive statistics, frequencies, and percentages. Univariable and multivariable logistic regression analyses were done to identify factors significantly associated with 30-day and frequent (more than two) readmissions. Adjustments were made to account for age, gender, race, diabetes, and transplant-related immunosuppressive medications. Results were presented as odds ratios (OR) with 95% confidence intervals (CI). A p-value <0.05 was considered statistically significant. All analyses were conducted using SPSS Statistics for Windows, version 23.0 (IBM, Armonk, NY).

## Results

A total of 931 patients who underwent solid organ abdominal transplants were included, and of these, 54% had undergone kidney transplants and 34% were liver transplants. The mean age of the patients was 50.4 ±12.8 years; 51% were males, and the majority were Caucasians (51%) followed by African Americans (41%). About a third (35%) of the patients had diabetes. Mycophenolate and tacrolimus were the most prescribed immunosuppressants at 95% and 93% respectively, with prednisone use at 89% and belatacept at just 23%.

Of note, 282 patients (30.3%) were readmitted within the first 30 days after discharge following transplants while 32.3% had multiple readmissions (two or more). On univariable analysis and multivariable analysis, after adjusting for age, gender, and race, diabetes (OR: 1.44, 95% CI: 1.03-2.01, p=0.029) and gastroparesis (OR: 2.58, 95% CI: 1.42-4.69, p=0.002) were significantly associated with higher odds of 30-day readmissions.

**Figure 1:**
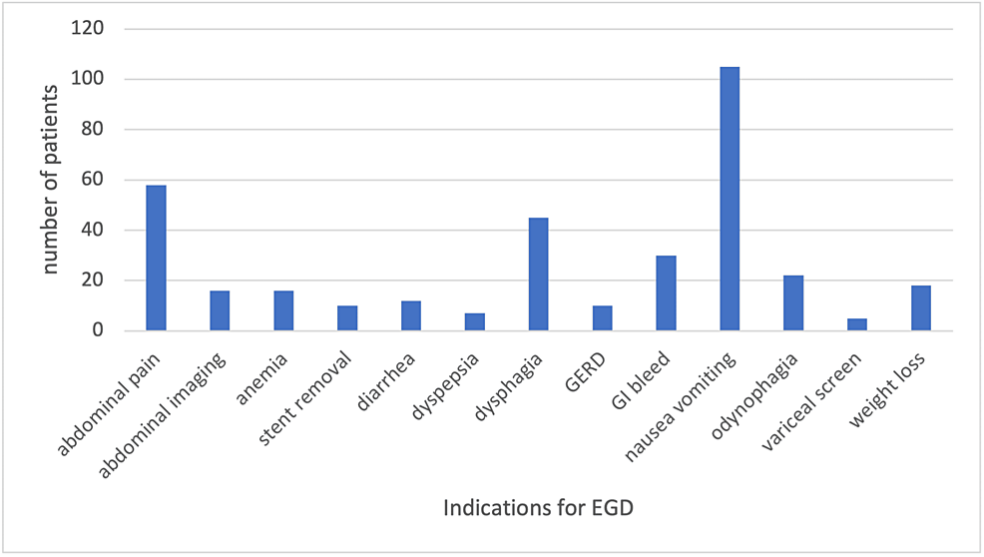
Indications or reasons for EGD* *The graph includes the most common indications for EGD. Indications seen less than five times are not included (bloating, cancer surveillance, early satiety, failure to thrive, PEG placement, and polyp surveillance) EGD: esophagogastroduodenoscopy; GERD: gastroesophageal reflux disease; GI: gastrointestinal; PEG: percutaneous endoscopic gastrostomy

**Figure 2:**
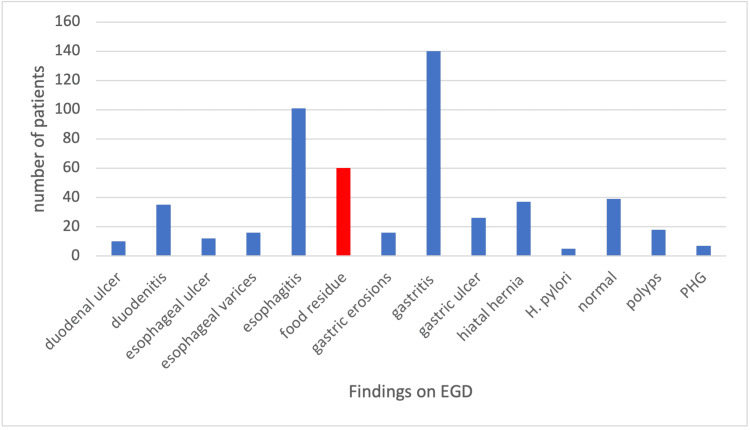
Findings on EGD* *Findings encountered less than five times not included (AVMs and masses) EGD: esophagogastroduodenoscopy; H. pylori: Helicobacter pylori; PHG: portal hypertensive gastropathy; AVM: arteriovenous malformation

A post-transplant upper endoscopy (EGD) was performed in 34% of the patients. Nausea/vomiting was the most common indication and gastritis was the most common finding (Figures 1, 2). Food residue was discovered on 19% of EGDs, suggesting gastroparesis. However, since only 22% of these patients had a further evaluation with a gastric emptying study, only 6% were formally diagnosed with gastroparesis since transplant. The presence of pre-transplant diabetes was significantly associated with higher odds of gastroparesis (OR: 5.17, 95% CI: 2.79-9.57, p<0.001).

Gastroparesis was also independently associated with higher odds for more than two readmissions (OR: 6.71, 95% CI: 3.45-13.1 p<0.001) while the use of belatacept (OR: 0.63, 95% CI: 0.42-0.94, p=0.023) was associated with a decrease in the odds of readmissions at 30 days (Table 1).

**Table 1:** Factors associated with outcomes of 30-day and two or more readmissions *Refers to multiple organs transplanted OR: odds ratio; CI: confidence interval

Variables	30-day readmissions, OR (95% CI) p-value	Two or more readmissions, OR (95% CI) p-value
Age	0.99 (0.99–1.01) p=0.739	0.99 (0.98–1.00) p=0.172
Caucasian	Reference	Reference
African American	1.18 (0.86–1.62) p=0.307	1.08 (0.79–1.48) p=0.644
Hispanic	0.60 (0.24–1.54) p=0.293	1.22 (0.54–2.74) p=0.635
Others	1.30 (0.44–3.88) p=0.632	0.96 (0.30–3.13) p=0.951
Female	Reference	Reference
Male	1.24 (0.92–1.68) p=0.154	0.85 (0.63–1.14) p=0.279
Diabetes	1.44 (1.03–2.01) p=0.029	1.15 (0.82–1.61) p=0.414
Hypothyroidism	1.07 (0.65–1.73) p=0.796	0.62 (0.37–1.06) p=0.082
Gastroparesis	2.58 (1.42–4.69) p=0.002	6.71 (3.45–13.10) p<0.001
Kidney transplant	Reference	Reference
Liver transplant	0.74 (0.51–1.08) p=0.119	1.15 (0.79–1.66) p=0.471
Multiple transplant*	1.26 (0.77–2.07) p=0.358	1.15 (0.68–1.92) p=0.603
Tacrolimus	1.02 (0.54–1.96) p=0.932	0.68 (0.37–1.24) p=0.211
Prednisone	0.99 (0.60–1.64) p=0.960	1.06 (0.65–1.74) p=0.810
Mycophenolate	1.02 (0.46–2.25) p=0.959	1.16 (0.53–2.54) p=0.710
Belatacept	0.63 (0.42–0.94) p=0.023	0.85 (0.57–1.26) p=0.418

 Table 2 illustrates the demographics and medical history of the patients.

**Table 2:** Demographics and medical history *Other or unknown. **Hemoglobin A1c within three months of transplant; ***Hemoglobin A1c: <7. ^α^Association with frequent readmission K: kidney; L: liver; SLK: simultaneous liver-kidney; SPK: simultaneous kidney-pancreas; P: pancreas; SD: standard deviation; N/A: unable to calculate due to single data point or not available; NS: not significant GN: glomerulonephritis; HCC: hepatocellular carcinoma; IS: immunosuppressive; MC: most common; Bela: belatacept; Tac: tacrolimus, MMF: mycophenolate mofetil; Pred: prednisone

			Composite (n=931)	K (n=506)	L (n=313)	SLK (n=36)	SPK (n=75)	P (n=1)	P-value^α^
Age in years (mean, SD)			50.39, 12.8	50.54, 13.2	52.1, 12.2	54.77, 11.3	40.6, 8.4	46, N/A	N/A
Percentage of female patients			49.3	50.2	46.3	47.2	56	100	N/A
Race (%)	Caucasian		51.3	37.1	71.6	61	58.7	100	N/A
	African American		41.1	53.8	21.1	36.2	38.7	0	
	Hispanic		3.2	4.2	2.7	0	1.3	0	
	Asian		2.2	2.6	2.3	0	0	0	
	Other*		2.2	2.3	2.3	2.8	1.3	0	
Pre-transplant A1c** (mean, SD)			8.75, 1.47	8.7, 1.64	8.03, 1.6	5.7, 0.88	9.01, 1.2	6.0, N/A	N/A
Diabetes			35	29.2	25.9	50	95	100	NS
DM controlled***			53.1	63.7	62.2	88.9	11.3	100	
DM uncontrolled			41.36	36.3	18.9	5.5	85.9	0	
Unknown			5.6	0	18.9	5.5	2.8	0	
Hypothyroidism (%)			10.1	9.3	7	19.4	22.7	22.7	NS
Indication for transplant		Most common	Diabetes	Hypertension	Viral hepatitis	Viral hepatitis	Diabetes	Diabetes	N/A
		Second	Hypertension	Diabetes	HCC	Diabetes			
		Third	Viral hepatitis	GN	Alcohol cirrhosis	Alcohol cirrhosis			
Discharge IS regimen			MC: Tac + MMF + Pred	MC: Tac + MMF + Pred	MC: Tac + MMF + Pred	MC: Tac + MMF	MC: Tac + MMF + Pred	Tac + MMF + Pred	N/A
			Second: Bela + Tac + MMF + Pred	Second: Bela + Tac + MMF + Pred	Second: Tac + MMF	Second: Tac + Pred	Second: Tac + MMF		
Food residue on EGD			60	27	20	2	11	0	N/A
Gastroparesis since transplant			52	24	10	0	18	0	<0.001

## Discussion

GI symptoms are commonly experienced in solid organ transplant recipients and can affect almost any part of the GI tract ranging from the mouth to the anus [1,3]. Studies have shown that the incidence of GI disorders is more common in patients who receive renal transplants than in the general population [4]. Furthermore, post-renal transplant dyspepsia not only worsens the quality of life but is also associated with an increased risk of graft failure. This in turn results in high morbidity and mortality in this patient population [5]. We aimed to evaluate upper GI disorders post-transplant at our institution to better characterize these associations.

Our study demographics demonstrated that there was a predominance of Caucasian men in their 50s with a past medical history of diabetes who underwent transplants and presented to the hospital for upper GI symptoms, which is similar to other studies in the literature.

The most common symptom prompting clinicians to conduct an EGD in our study was the presence of nausea and vomiting followed by abdominal pain and dysphagia. These symptoms may be due to the numerous immunosuppressive medications many patients take on a daily basis, such as mycophenolate mofetil (MMF). Significantly, nausea, vomiting, dyspepsia, and anorexia have been found to be frequent in patients who are on MMF [6]. Based on our findings, 95% of patients were discharged home with MMF as part of their immunosuppression regimen, which may play a significant role in the development of their upper GI symptoms leading to frequent readmissions post-transplant. Although, on multivariable analysis, this was not an independent predictor for readmissions. Analysis of data from a retrospective study from 10 transplant centers demonstrated that 49.7% of patients experienced at least one GI complication within the first six months post-transplant, with 66.8% of these having multiple GI complications. Of the patients with GI complications, 39% experienced MMF dose adjustments or discontinuation of MMF therapy. It was also found that the patients with GI complications who experienced MMF dose adjustments/discontinuation had a significantly higher incidence of acute rejections compared with patients without GI complications because of either subtherapeutic dosing or impaired clinical outcomes [7]. Identifying other modifiable factors rather than concentrating on MMF dose adjustments to alleviate GI symptoms may potentially be advantageous for post-transplant patients. For example, multiple reports on patients post-solid organ transplant have shown that conversion from MMF to enteric coated-mycophenolate sodium (MPS) was associated with a significant improvement in GI symptoms and health-related quality of life in both kidney- and liver-transplant recipients [8,9].

Findings from EGDs in our study indicate that the most common finding in patients with dyspepsia was gastritis followed by esophagitis, which is in keeping with what is found in the literature [10]. However, food residue was found in approximately 19% of EGDs, suggesting that these patients could have gastroparesis; however, since only 22% of these patients had a further evaluation with a gastric emptying study, only 6% were formally diagnosed with gastroparesis since transplant. While evidence has shown that patients with a diagnosis of gastroparesis prior to transplant may have improvement with kidney and or pancreas transplant, gastroparesis remains a significant problem for post-transplant patients [11]. Our study shows that the odds of these patients having greater than two admissions are six times more than those without a diagnosis of gastroparesis.

Diabetes mellitus and diabetic nephropathy remain the most common indications for solitary kidney as well as kidney-pancreas transplants worldwide, and gastroparesis is a commonly associated disorder in this patient population. Our results suggest that patients with kidney or kidney-pancreas transplants are more likely to develop gastroparesis based on the number of patients who were diagnosed with gastroparesis post-transplant (Table 2), which may be explained by the fact that these patients may have undergone transplants initially due to complications of diabetes. While this may be the case, gastroparesis has been described in patients without diabetes after solid organ transplants, such as in those who underwent lung and heart transplantation. This may lead to impairment in the pharmacokinetics of immunosuppressive medication, which leads to an increased risk of severe side effects in these patients [12]. The pathophysiology of post-transplantation gastroparesis is not completely understood and is likely to be multifactorial in nature. However, there is some evidence to suggest that post-lung and heart transplant gastroparesis may be related to injury to the vagus nerve [13]. Nonetheless, the data in Table 1 suggests that the presence of pre-transplant diabetes was significantly associated with higher odds of gastroparesis and was in turn independently associated with higher odds of 30-day readmissions (OR: 1.44, 95% CI: 1.03-2.01, p=0.029).

GI complications remain a significant challenge in patients who undergo solid organ transplants, thereby contributing to substantial morbidity and mortality. While there are multiple cofounders that may contribute to this, such as race, age, and comorbidities, it is important that physicians remain vigilant in recognizing and treating associated disorders such as diabetes and gastroparesis due to the significant effect they have on the quality of life and the negative impact on clinical outcomes in these patients [2].

Certainly, there are some limitations to this study. This was a retrospective, single-center, cross-sectional study. Therefore, no follow-up on responses to specific therapies for GI symptoms was available. Additionally, our study did not include all GI complications such as diarrhea and constipation. However, our focus was on understanding the extent to which patients have upper GI symptoms that would impact the ability to tolerate their medications as well as maintain adequate nutrition and hydration. The effects of the dosing of different immunosuppressive medications were also not considered. However, this can be evaluated in the future to determine if certain dosing regimens are better tolerated by this cohort. Indeed, there are several strengths to our analysis, such as having a large cohort available to evaluate. This enabled us to make important associations regarding the risks for post-transplant dyspepsia and highlight the importance of identifying the presence of gastroparesis, which may be effectively managed by lifestyle modifications, antiemetics, and prokinetics.

## Conclusions

In summary, following abdominal solid organ transplants, a significant number of patients were readmitted for dyspeptic symptoms. The presence of diabetes and gastroparesis was significantly associated with higher odds of readmissions while the use of belatacept was associated with lower readmission rates. Close follow-up following solid organ transplants is necessary to screen for these symptoms and provide early treatment to reduce the rates of readmissions and poor clinical outcomes.

## Appendices

**Table 3:** Supplemental table – univariate logistic regression *Refers to multiple organs transplanted OR: odds ratio; CI: confidence interval; MMF: mycophenolate mofetil

Variables	30-day readmissions, OR (95% CI) p-value	Two or more readmissions, OR (95% CI) p-value
Age	1.00 (0.99–1.01) p=0.584	0.99 (0.98–1.00) p=0.021
Caucasian. Female. Kidney transplant	Reference	Reference
African American	1.17 (0.88–1.56) p=0.271	1.06 (0.80–1.40) p=0.688
Hispanic	0.59 (0.24–1.47) p=0.258	1.11 (0.51–2.41) p=0.801
Others	1.54 (0.55–4.39) p=0.413	1.05 (0.35–3.09) p=0.933
Diabetes	1.80 (1.35–2.40) p<0.001	1.30 (0.98–1.73) p=0.072
Hypothyroidism	1.15 (0.73–1.81) p=0.550	0.78 (0.49–1.26) p=0.308
Gastroparesis	3.12 (1.77–5.50) p<0.001	7.06 (3.71–13.5) p<0.001
Liver transplant	0.74 (0.55–1.00) p=0.054	1.02 (0.76–1.36) p=0.905
Multiple transplant*	2.05 (1.38–3.06) p<0.001	1.49 (1.00–2.22) p=0.052
Tacrolimus	1.12 (0.64–1.97) p=0.692	0.78 (0.46–1.33) p=0.364
Prednisone	1.11 (0.70–1.75) p=0.654	1.05 (0.67–1.63) p=0.833
MMF	1.05 (0.53–2.08) p=0.897	0.92 (0.48–1.79) p=0.811
Belatacept	0.72 (0.51–1.01) p=0.059	0.85 (0.61–1.19) p=0.353
Male	1.20 (0.91–1.59) p=0.198	0.83 (0.63–1.09) p=0.179
